# Minireview: algal natural compounds and extracts as antifoulants

**DOI:** 10.1007/s10811-017-1322-0

**Published:** 2017-11-06

**Authors:** Mahasweta Saha, Franz Goecke, Punyasloke Bhadury

**Affiliations:** 1 Benthic Ecology, Helmholtz Center for Ocean Research, Düsternbrooker weg, 24105 Kiel, Germany; 2 Present Address: School of Biological Science, University of Essex, Colchester, CO 43 SQ, UK; 3 Department of Plant and Environmental Science (IPV), Norwegian University of Life Sciences (NMBU), Ås, Norway; 4 Integrative Taxonomy and Microbial Ecology Research Group, Department of Biological Sciences, Indian Institute of Science Education and Research Kolkata, Mohanpur, Nadia, West Bengal 741246 India

**Keywords:** Algae, Biofouling, Natural products, Antifouling defense, Antibacterial, Anti-diatom, Anti-macrofouling

## Abstract

Marine biofouling is a paramount phenomenon in the marine environment and causes serious problems to maritime industries worldwide. Marine algae are known to produce a wide variety of chemical compounds with antibacterial, antifungal, antialgal, and anti-macrofouling properties, inhibiting the settlement and growth of other marine fouling organisms. Significant investigations and progress have been made in this field in the last two decades and several antifouling extracts and compounds have been isolated from micro- and macroalgae. In this minireview, we have summarized and evaluated antifouling compounds isolated and identified from macroalgae and microalgae between January 2010 and June 2016. Future directions for their commercialization through metabolic engineering and industrial scale up have been discussed. Upon comparing biogeographical regions, investigations from Southeast Asian waters were found to be rather scarce. Thus, we have also discussed the need to conduct more chemical ecology based research in relatively less explored areas with high algal biodiversity like Southeast Asia.

## Introduction

Marine biofouling—the colonization and growth of micro- and macro-organisms on any submerged surface (living and man-made structures) is paramount in the marine environment (Wahl 1989). An average milliliter of natural seawater contains 10^6^ cells of bacteria, 10^3^ microalgae, and 10^2^ propagules of algae and benthic invertebrate larvae (Harder 2009) making any undefended surface quite likely to be colonized by micro- and macro-foulers. Uncontrolled biotic coverage is heavily detrimental for efficient operation and functioning of such submerged man-made structures (reviewed in Maréchal and Hellio 2009; Callow and Callow 2011). Biofouling on structures like ships not only increase ownership costs but are also accompanied with environmental pollution through increased emission of gases like carbon dioxide, carbon monoxide, and sulfur dioxide (Chambers et al. 2006) and is also involved with transport of invasive species (Gollasch 2002). Remediation of biofouling on ship hulls only costs approximately € 120 billion per year (Chapman et al. 2014). To counter such detrimental effects, biofouling was previously controlled using toxic antifouling coatings. While the use of antifouling compounds like tributlyl tin (TBT) and copper oxide have been found to be the most effective method, their non-targeted effect on other marine organisms like toxicity, imposex, bioaccumulative effect, and contamination of the food chain (Fernández-Alba et al. 2002; Bellas 2006;) has led to a total ban of TBT-based coatings in 2008 by the International Maritome Organization. Although new biocide formulations like irgarol, chlorothalonil, dichlofluanid, and diuron were introduced in marine antifouling AF coatings and initially thought to be environment friendly, later they were reported to be toxic, accumulating in marinas and harbors (Chapman et al. 2014). The ban of TBT and other toxic AF coatings has drawn a huge amount of interest towards bio-inspired AF approaches, i.e., the investigation of novel, environment-friendly AF compounds from organisms that are mostly free from foulers colonizing their surfaces. Latest strategies towards development of environment-friendly AF coatings included biomimetic approaches, “biomimicry” (reviewed by Scardino and de Nys 2011), and incorporation of natural antifouling compounds into marine paints (Chambers et al. 2006). However, for their potential application in industries, such compounds must be cost effective in terms of production, long lasting, easy to use, and non-toxic to marine biota (Ralston and Swain 2009). Efforts made and the systems tested till date have not been enough successful as they failed to meet all or partly the criteria stated above since such compounds are usually not stable once exposed to the natural environment (Maréchal and Hellio 2009); making them durable is a current challenge. Also, development of a natural product is time consuming and a rigorous process. Further, their non-toxicity towards non-target species needs to be assured before being deployed in the field.

Benthic marine environments are quite diverse and characterized by extreme competition for light, space, nutrient, and other resources (Wahl 2009). Space being a limiting factor all potential benthic organisms are also confronted and colonized by micro and macro settlers—a phenomenon typically termed as epibiosis when the substrate involved is a living one (Wahl 1989). As epibiosis is usually detrimental to the (basibiont) host (Wahl 2008), many basibionts have developed either a physical or chemical (or both) protection mechanism against such colonizers, either by themselves (reviewed by Da Gama et al. 2014) or through symbiotic relationships with bacterial epibionts (reviewed by Singh et al. 2015). Over the past 25 years, several investigations have reported many natural antifouling compounds being extracted and characterized from varied marine prokaryotes and eukaryotes in assays against relevant fouling organisms and a number of excellent reviews reporting their potential use as antifouling compounds have been published (reviewed by Fusetani 2011; Dobretsov et al. 2013; Puglisi et al. 2014; Qin et al. 2013).

Being sessile and restricted to the photic zone, macroalgae offer optimal growth conditions to many epibiotic organisms (Harder 2008). Their three-dimensional structure also offers a large surface area for settlement in benthic marine habitats (Seed 1985). Thus, macroalgae are highly susceptible to epibiosis in comparison to other potential basibionts. Macroalgae have evolved efficient defense mechanisms as a mean of protection and thus have been found to be producers of wealth of antifouling compounds (reviewed by Bhadury and Wright 2004; Da Gama et al. 2014). Unlike macroalgae, microalgae are not susceptible to colonization by macro-epibionts, but they also experience intense competition for space and other resources with their neighbors. Certain microalgae are known to control their microenvironment through the employment of allelochemicals which have been suggested to be responsible for observed patchy distribution in species composition around these microalgae (Saburova et al. 1995; Borowitzka 2016). Such synergistic and antagonistic ecological interactions through employment of chemicals have triggered interest among marine natural product chemists leading to the examination of numerous natural products as a possible basis for novel anti-biofilm compounds like cyanogen bromide produced by *Nitzschia* cf. *pellucida* (Vanelslander et al. 2012).

In the last 5 years, a large number of macroalgal species (89 species in total) and few microalgal species (13 species in total) have been tested for antifouling activity and several metabolites have been isolated with related bioactivity. The scope of the current review is to cover their activity against maritime fouling and not medical and industrial fouling. In this review, we have summarized the current status of marine algal antifouling compounds and extracts isolated and identified between January 2010 and June 2016. We also present current challenges and future perspectives on development of antifouling strategies from marine algae. Currently, there is an important amount of information on metabolic routes, physiological responses, and algal cultivation, and there are an increasing number of studies on algal genomics (Radakovits et al. 2010; Ai et al. 2015; Hlavová et al. 2015; Perez Garcia and Bashan 2015) which, when grouped together, we consider essential for the development of algal biotechnology. For a potential sustainable development of natural algae-related antifouling strategies, we have two main focuses: (a) the use of available and upcoming genomic information to understand biofouling from the algal genomic perspective (we present the case of diatom genes linked to biofilm formation) and thereby develop gene knock-out-based (or modification) antifouling strategies and (b) it is necessary to couple screening, isolation of compounds, and the genomic studies with industrial upscaling and metabolic engineering for a sustainable commercial production of natural micro algal compounds. Also, summing up all the ecologically and industrially relevant antifouling investigations, it is noteworthy that the majority of investigations have focused on algae from temperate waters despite the fact that tropical waters are rich in biodiversity. Therefore, in this review, we have highlighted the importance of expanding the area of sampling to still un-explored zones (we present Southeast Asia as a case study) with chemical ecology research objectives, i.e., isolating and testing natural compounds in ecologically relevant bioassays.

## Antifouling compounds from macroalgae

**Fig. 1:**
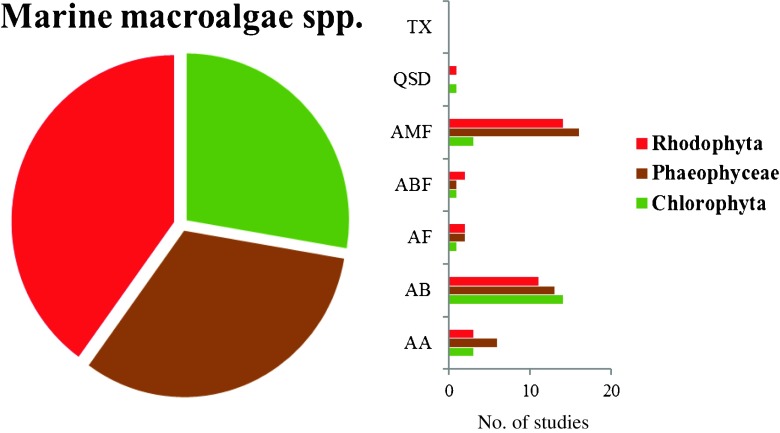
Bioactivity of metabolites or extract fractions from a total of 24 species of red algae, 13 species of green algae, and 18 species of brown algae, active against marine relevant species during the period 2010–2016. Antibacterial activity (AB), antifungal (AF), anti-microalgal including diatoms and cyanobacteria (AA), anti-macrofouling including mollusks and crustacean (AMF), quorum sensing disruptor (QSD), toxins (TX), and anti-biofilm (ABF)

Apart from being the major primary producers in temperate ecosystems and the largest biomass producers in marine environment, macroalgae produce a diverse array of natural compounds as mode of protection against natural enemies (Goecke et al. 2010). Over the last 5 years, a number of studies (some of which are discussed below) have reported new antifouling compounds from macroalgal extracts like the cystophloroketals and chromanols, along with an increasing number of reports of activities from crude extracts of different polarities (Table 1, Fig. 1).

**Table 1:** Antifouling compounds and extracts from macroalgae

Source	Biogenic compound(s)/type of extract	Bioactivity	Origin	Reference
*Asparagopsis taxiformis* ^RHO^	Mahorone 5-bromomahorone^1^	AB	Mayotte	Greff et al. 2014
*Asparagopsis taxiformis* ^RHO^	Methanolic extract	AB, QSD	India	Jha et al. 2013
*Bonnemaisonia hamifera* ^*RHO*^	1,1,3,3-Tetrabromo-2-heptanone^2^	ABF	Sweden	Persson et al. 2011
*Caulerpa prolifera* ^CHL^	Ethanol and methanol extracts	AB	Brazil	Silva et al. 2013
*Ceramium botryocarpum* ^RHO^	Ethanol fraction	AA	France	Silkina et al. 2012
*Ceramium rubrum* ^RHO^	Dichloromethane extract	AF	Chile	Cortés et al. 2014
*Chondrus crispus* ^RHO^	Toluene-soluble crude ethanolic extract	ABF	Ireland	Salta et al. 2013
*Chondrus crispus* ^RHO^	Crude ethanol extracts	AA, AB	Ireland	Chambers et al. 2011
*Cladophora clavuligera* ^CHL^	Methanolic and dichloromethane extract	AB	India	Bragadeeswaran et al. 2011a
*Cystoseira tamariscifolia* ^PHE^	Cystophloroketals A-B	AA, AB, AF	Algeria	Hattab et al. 2015
*Cystoseira tamariscifolia* ^PHE^	monocyclic meroditerpenoid	AMF	Algeria	Hattab et al. 2015
*Dictyota spp.* ^*PHE*^	Diterpenes, glicerol derivatives	AB, AF	France	Othmani et al. 2014
*Dictyota fasciola* ^PHE^	Dichloromethane and methanol extract	AA, AB, AMF	Tunisia	Ktari et al. 2010
*Dictyosphaeria ocellata* ^CHL^	Methanol extract	AB, ABF	USA	Sneed and Pohnert 2011a, b
*Fucus vesiculosus* ^PHE^	Fucoxanthin^3^, dimethyl sulphopropionate^4^, proline^5^	AB, ABF	Germany	Saha et al. 2011, 2012, Wahl et al. 2010, Lachnit et al. 2013
*Gracilaria edulis* ^RHO^	Isoamyl alcohol extract	AB	India	Rajan et al. 2015
*Hypnea musciformes* ^RHO^	Ethanol and methanol extracts	AB	Brazil	Silva et al. 2013
*Laurencia johnstonii* ^RHO^	Ethly ether extract	AA, AB, AF	Mexico	Águila-Ramírez et al. 2012
*Laurencia* sp.^RHO^	Omaezallene^6^ intricatetraol^7^	AMF	Japan	Umezawa et al. 2014
*Laurencia translucida* ^RHO^	Fatty acid derivatives	AMF	Brazil	Paradas et al. 2016
*Laurencia viridis* ^RHO^	28-Hydroxysaiyacenol B Saiyacenol C 15,16-epoxythyrsiferol A 15,16-Epoxythyrsiferol B	AMF	Spain	Cen-Pacheco et al. 2015
*Padina gymnospora* ^PHE^	Ethanol and methanol extracts	AB	Brazil	Silva et al. 2013
*Sargassum horneri* ^PHE^	Chromanols ^8^	AA, AB, AMF	South Korea	Cho 2013
*Sargassum muticum* ^PHE^	Galactoglycerolipids	AB, AF, AMF	France	Plouguerné et al. 2010b
*Sargassum muticum* ^PHE^	Ethanol fraction	AA	France	Silkina et al. 2012
*Sargassum polyceratium* ^PHE^	Hexane extract	AB	Martinique	Thabard et al. (2011)
*Sargassum wightii* ^PHE^	Methanol extract	AB	India	Bragadeeswaran et al. 2011b
*Sargassum vulgare* ^PHE^	Unidentified polar compounds	AMF	Brazil	Plouguerné et al. 2012
*Sargassum vulgare* ^PHE^	Hexane extracts methanol and dichloromethane extracts polyphenolic extracts	AA, AB, AMF	Brazil	Plouguerné et al. 2010a
*Sphaerococcus coronopifolius* ^RHO^	Bromosphaerol^9^	AMF	Greece	Piazza et al. 2011
*Taonia atomaria* ^PHE^	sesquiterpenes polyunsaturated fatty acids	AB, AMF	France	Othmani et al. 2015
*Ulva fasciata* ^CHL^	Ethanol and methanol extracts	AB	Brazil	Silva et al. 2013
*Ulva intestinalis* ^CHL^	Hexane extract	AB	Thailand *	Srikong et al. 2015
*Ulva lactuca* ^CHL^	Ethyl ethanol extract	AA, AB, AF	Mexico	Águila-Ramírez et al. 2012
*Ulva pertusa* ^CHL^	Alkaloids, phenolic acid	AA	China	Sun et al. 2015
*Ulvaria obscura* ^CHL^	Dopamine	AA, AMF	USA	Van Alstyne et al. 2014
8 spp. macroalgae^CHL,PHE,RHO^	Methanol extract	AB	Malaysia*	Natrah et al. 2015
11 spp. macroalgae^CHL,PHE,RHO^	Polar and non-polar extracts	QSD	Brazil	Batista et al. 2014
30 spp. macroalgae^CHL,PHE,RHO^	Diverse aqueous and organic extracts	AMF	France	Maréchal and Hellio 2011

**Fig. 2:**
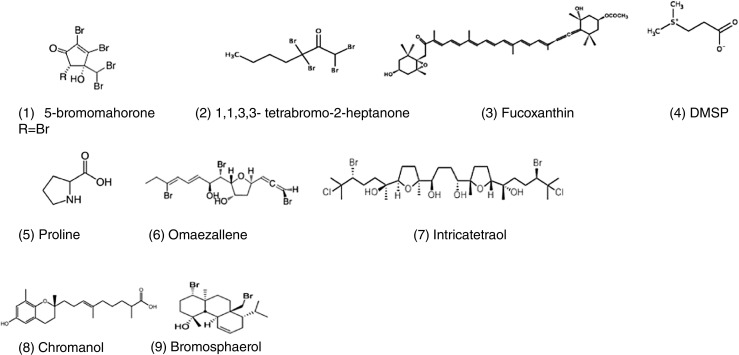
Selected antifouling compounds from macroalgae

The bioactivities are the following: antibacterial activity (AB), antifungal (AF), anti-microalgal including diatoms and cyanobacteria (AA), anti-macrofouling including mollusks (AMF), quorum sensing disruptor (QSD), and anti-biofilm (ABF). Also the algae groups: Chlorophyta (CHL), Phaeophyceae (PHE), and Rhodophyta (RHO). Studies made in Southeast Asia are highlighted with an asterisk. 1–9 = compounds listed in Fig. 2

Certain genera, like the red algae *Asparagopsis* and *Laurencia*, and the brown alga *Sargassum* continue to be source of new active antifouling compounds. *Asparagopsis* spp. belong to the order Bonnemaisoniales, which is known to be a rich source of halogenated bioactive compounds (Jha et al. 2013). Recent study of the introduced red alga *Asparagopsis taxiformis* collected from the Indian Ocean has led to the discovery of two new highly brominated cyclopentenones: mahorone and 5-bromomahorone with antimicrobial activity against both marine and terrestrial microbes (Greff et al. 2014). Another investigation on the same species, collected from the intertidal region of the Arabian Sea and Bay of Bengal, has found the methanolic extract to have anti-quorum sensing activity (Jha et al. 2013). Other seaweed species have also been shown to produce quorum-sensing inhibitors (Carvalho et al. 2017). Members of the genus *Laurencia* (Ceramiales) are also known to be a rich source of halogenated secondary metabolites (Blunt et al. 2009) and they continue to be source of new compounds. Recently, a new omaezallene and four new polyether triterpenoids with anti-macrofouling activity have been discovered from *Laurencia* sp. and *Laurencia viridis*, respectively (Umezawa et al. 2014; Cen-Pacheco et al. 2015).

Brown algae of the genus *Cystoseira* (Fucales) are known to be good source of bioactive terpenoid derivates, specially meroterpenoids and diterpenoids (reviewed by Gouveia et al. 2013). A monocyclic meroditerpenoid isolated from *Cystoseira tamariscifolia* has shown high potential for inhibition of common foulers like *Balanus amphitrite* and *Mytilus edulis*. Cystophloroketals A and B from the same alga have shown high inhibition activity against two species of fouling microalgae and moderate antimicrobial activity against a range of bacteria, fungi, and microalgae (Hattab et al. 2015). Along with the reported role of pholorotannins as antifoulants from certain fucoid species, certain metabolites like fucoxanthin and dimethylsulfoniopropionate (DMSP), which were previously considered to have just primary functions, have been reported to function as regulators of the microbial density and composition over *Fucus vesiculosus* (Lachnit et al. 2013; Saha et al. 2011, 2012, 2014). Identified compounds with AF activity are provided in Fig. 2.

Furthermore, many other studies have investigated the activities of both polar and non-polar extracts and reported antibacterial, antimicroalgal, and antifungal properties from a variety of algae belonging to the genera *Caulerpa*, *Chondrus*, *Dictyota*, *Padina*, *Sargassum*, and *Ulva* (Chambers et al. 2011; Cho 2013; Ktari et al. 2010; Silva et al. 2013, see Table 1).

In terms of number of species, more than a quarter of the studies involved red macroalgae and the remaining two third was composed of brown and green seaweeds (Fig. 1). In terms of the chosen bioactivity, most of the studies demonstrated an antimacrofouling activity (e.g., Maréchal and Hellio 2011; Van Alstyne et al. 2014) followed by antibacterial (e.g., Silva et al. 2013; Natrah et al. 2015) and antimicroalgal activity (e.g. Silkina et al. 2012; Sun et al. 2015). This may be based on the fact that, space being limited in the benthic environment, macrofoulers usually tend to settle on macroalgal surfaces thereby selecting for antimacrofouling defenses. Also, epibacterial colonization is ubiquitous on algal surfaces with the ability to negatively impact macroalgal fitness (Wahl et al. 2012) thereby selecting for antibacterial defenses.

## Antifouling compounds from dinoflagellates, diatoms, and other marine microalgae

**Fig. 3:**
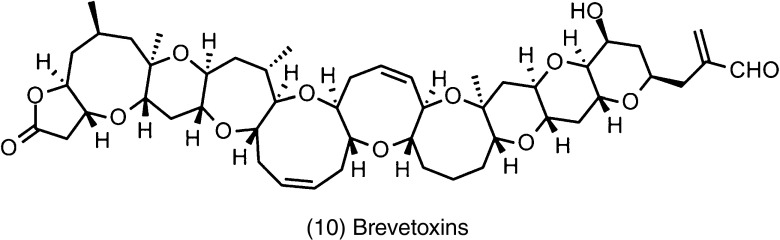
Brevetoxins from microalgae *Karenia brevis*

Several studies have been conducted to investigate the products of microalgal metabolism, not only to understand its nature but also to search for substances with possible applications to humans in different fields of interest (de De Morais et al. 2015). Some of the earliest and most extensive research on microalgal secondary metabolites has been on cyanobacteria, dinoflagellates (Dinophyceae), a few other microflagellates, and diatoms (Bacillariophyceae) (Garcia Camacho et al. 2007). This is because of the ability of certain bloom-forming species to produce structurally quite diverse toxins like brevetoxins produced by *Karenia brevis* (Fig. 3). Such bloom phenomena by toxic microalgae are generally known as harmful algal blooms (HABs). HABs can be highly toxic to humans and other animals and have further dramatic health and socio-economic impacts (Pearson et al. 2010; Clément et al. 2016; Mazard et al. 2016).

Different species from divergent phylogenetic orders of the dinoflagellates produce phytotoxins, e.g., *Alexandrium* spp. and *Protoceratium* spp. (Gonyaulacales), *Azadinium* sp. (Dinophyceae incertae sedis), *Dinophysis* spp. (Dinophysiales), *Gymnodinium* spp. and *Karenia* spp. (Gymnodiniales), and *Prorocentrum* spp. (Prorocentrales), and diatoms as well as *Pseudonitzschia* spp. (Bacillariales). Such diversity is magnified at a rich subspecies level, and in consequence, the heterogeneity of associated phycotoxins and its derivatives are quite variable and big (Orr et al. 2013). The production of such toxins has been seen as an ecological advantage since the release of those chemicals into the environment may deter, inhibit growth, or kill competing species and predators (Caroppo and Pagliara 2011; Ma et al. 2011; Poulson-Ellestad et al. 2014; Rolton et al. 2014; Bagwell et al. 2016). Allelopathic interactions between microalgal species also may contribute to the formation and succession of red tides (Cai et al. 2014). As the interactions with many potential foulers are regulated by chemical cues, there is a high potential in the identification and use of such compounds for biotechnological applications like antifouling compounds.

With respect to other microalgae groups, in the last decade, most of the studies had focused on the content of fatty acids and pigments aiming for their use in biotechnology, e.g., carotenoids and polyunsaturated fatty acids (PUFAs) for feed, and as active ingredients for cosmetics, among others (Abida et al. 2013; Borowitzka 2013a). Even though green microalgae are generally regarded safe for human consumption, there is still a knowledge gap in understanding the metabolic and biochemical potential of these algae (Bagwell et al. 2016).

Between January 2010 and June 2016, only 14 relevant studies were conducted on marine microalgal species, which tested extracts against other marine organisms. Only few compounds (e.g., fatty acids) were isolated from diatoms and dinoflagellates which have demonstrated antimicrobial or antifouling activities (Desbois et al. 2010). Although the number of isolated compounds is clearly less than in cyanobacteria (data not shown), the potential exists to increase that number, because most of these studies have reported bioactivity of diverse cultures and their organic extracts but did not isolate the active compounds as yet (Table 2).

**Table 2:** Antifouling compounds and extracts from dinoflagellates and diatoms

Source	Biogenic compound(s)/ type of extract	Bioactivity	Origin	Reference
*Alexandrium tamarense* ^DF^	Lipidic extracts	AA	Scotland	Ma et al. 2011
*Amphora* cf. *capitellata*^DI^	Ethanol extract	AF	Turkey	Montalvaõ et al. 2016
*Isochrysis galbana* ^HA^	Fatty acids	AB	Isle of Man	Molina-Cárdenas et al. 2014
*Karenia brevis* ^DF^	Culture extracts, 6 unidentified compounds	AA	USA	Poulson et al. 2010
*Karenia brevis* ^DF^	Diverse metabolites	AA	USA	Prince et al. 2010
*Karenia brevis* ^DF^	Culture extracts, unidentified compounds	AA	USA	Poulson-Ellestad et al. 2014
*Karenia brevis* ^DF^	Culture extracts, brevetoxins^10^	AMF, TX	USA	Rolton et al. 2014
*Lingulodinium polyedrum* ^DF^	Diverse extracts	AB	Mexico	Quijano-Scheggia 2016
*Nitzschia communis* ^DI^	Ethanol extract	AB	Turkey	Montalvaõ et al. 2016
*Odontella aurita* ^DI^	Ethanol extract	AB	India	Hemalatha et al. 2016
*Ostreopsis* cf. *ovata*^DF^	Culture extracts	AMF, TX	Italy	Caroppo and Pagliara 2011
*Ostreopsis* cf. *ovata*^DF^	Palytoxin-like compounds	AMF, TX	Italy	Gorbi et al. 2012
*Phaeodactylum tricornutum* ^DI^	Culture extracts	AA	China	Cai et al. 2014
*Prorocentrum donghaiense* ^DF^	Culture extracts	AA	China	Cai et al. 2014
*Protoceratium reticulatum* ^DF^	Culture extracts	AA	Greenland	Sala-Pérez et al. 2016
*Skeletonema marinoi* ^DI^	Acetonic extracts	AB	Italy	Lauritano et al. 2016
*Thalassiosira rotula* ^DI^	Diverse extracts	AB	Australia	Qin et al. 2013

The bioactivities are the following: antibacterial activity (AB), antifungal (AF), anti-macroalgal (AM), anti-microalgal including diatoms and cyanobacteria (AA), anti-macrofouling including mollusks (AMF), quorum sensing disruptor (QSD), anti-biofilm (ABF), and toxins (TX). Also the algae groups: diatoms (DI), dinoflagellates (DF), and haptophytes (HA). 10 = compound listed in Fig. 3

**Fig. 4:**
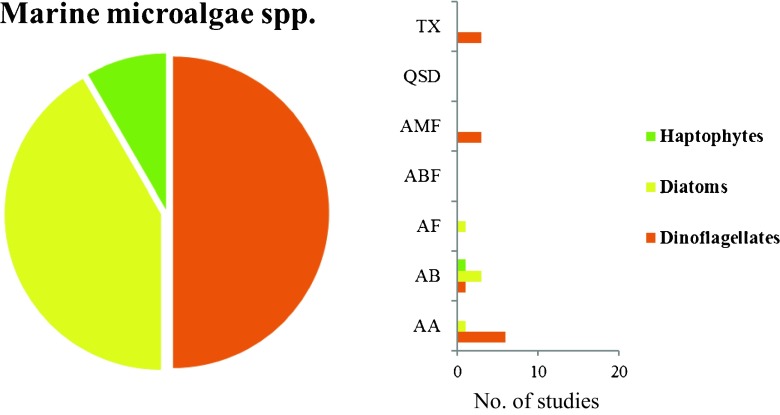
Bioactivity of metabolites or extracts fractions from a total of six species of dinoflagellates, five species of diatoms and one haptophyte, active against marine relevant species during the period 2010–2016. Antibacterial activity (AB), antifungal (AF), anti-microalgal including diatoms and cyanobacteria (AA), anti-macrofouling including mollusks and crustacean (AMF), quorum sensing disruptor (QSD), toxins (TX), and anti-biofilm (ABF)

In terms of number of species, half of the studies involved dinoflagellates and most of the second half was composed of diatoms. Only one study incorporated a marine haptophyte, and thus, many other microalgal groups remain unstudied (Fig. 4). In terms of the chosen bioactivity, most of the studies demonstrated an antialgal activity (Fig. 4). The latter may be based on the fact that the production of antialgal metabolites would be an ecological advantage in a specific niche and/or space. Furthermore, organisms like mollusks and crustaceans not only compete with benthic microalgae for space, but are also important microalgal consumers, with the ability to affect microalgal populations.

## A case study: antifouling compounds from Southeast Asia

Southeast Asia has an outstanding species richness and endemism. It has an extensive coastline (e.g., Indonesia, 81.000 km; Vietnam, 3.260 km) with many islands and rivers flowing into the extended and large continental shelf (Aungtonya and Liao 2002; Gerung et al. 2006; Soe-Htun et al. 2009; Phang et al. 2015). This produces a diverse variety of ecosystems, ranging from extensive lagoons, estuaries, and mangroves, to rocky shores and coral reefs, which provide suitable habitats for luxuriant algal growth (Nguyen et al. 2013). In fact, the triangle formed by the Malay Peninsula, The Philippines, plus New Guinea (“The Coral Triangle”) is recognized as a global biodiversity hotspot where most tropical marine groups have their greatest diversity of species (Todd et al. 2010; Selig et al. 2014). Researchers have noticed that a high diversity of species may be translated in a higher diversity of secondary metabolites. But not only that, those species may harbor silent gene clusters coding the production of “new” metabolites, which may be expressed under different environmental conditions (Bode et al. 2002; Brakhage and Schroeckh 2011). Thus, biogeography may multiply this number and even enhance bioactivity of such metabolites.

The exact number of macro- and micro-algal species for Southeast Asia is still unknown and there are many species yet to be discovered (Kawaguchi and Hayashizaki 2011). Research has mostly focused on economic resources, as several macroalgae are important economic resources in Southeast Asia (S.E.A.). Thus, the existing coverage of species is conspicuous by the omission of small groups (Wafar et al. 2011), or other “less important” economic species, and therefore, we understand little of their distributions and potential uses (Webb et al. 2010). Given the unlocked potential of algal compounds from S.E.A, in this review, we have highlighted the importance of exploring this area with more depth.

### Algal compounds in general from S.E.A

**Fig. 5:**
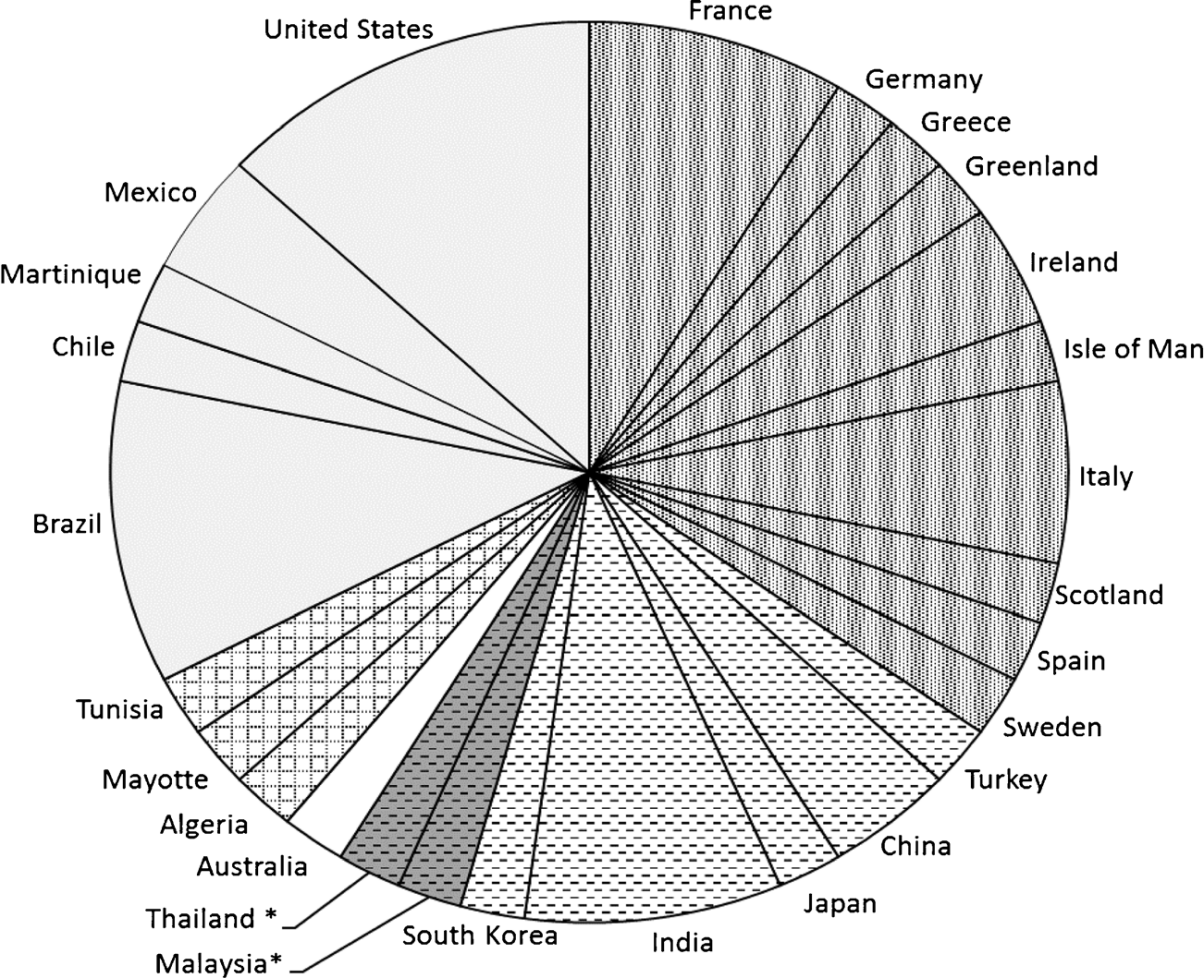
Studies conducted between January 2010 and June 2016 based on geographical locations. % by zone = America 32%, Europe 34%, Africa 6%, Oceania 2%, Asia 23%, S.E.A. 4% (of the 23% of Asia)

If we compare the collection effort globally of all type of organisms, the S.E.A region has been well explored and reporting of compounds from these regions has been rapidly accelerating since 1990 (Blunt et al. 2016). According to Blunt et al. (2016), maritime Southeast Asia (and Papua New Guinea) has produced in 50 years 1340 compounds as reported in 502 publications, plus the mainland of Southeast Asia (including East Malaysia) has produced 457 compounds in 173 publications. However, considering the information in the excellent reviews of new marine natural products published by Blunt et al. (2011, 2012, 2013, 2014, 2015, 2016), between the years 2008 to 2014, the contribution of compounds isolated from marine algae species growing in S.E.A is limited to only 29 compounds, from which only 23 are newly described for science (Table 3), when compared to the compounds described from algae collected worldwide. It is also interesting to note that the investigations were mostly limited to red algae of the genus *Laurencia* (Table 4). When we compare the total amount of new natural compounds reported from marine algae worldwide, the yield of natural compounds from marine algae reported from Southeast Asia is not significant (Fig. 5), contradicting the great diversity of the area.

**Table 3:** Detail of the yearly new identified natural compounds isolated from different marine algae groups (data based on Blunt et al. 2016). The contribution of compounds isolated from species growing in Southeast Asia (S.E.A) is specified

	New identified compounds													
Organisms	2008 total	2008 SEA	2009 total	2009 SEA	2010 total	2010 SEA	2011 total	2011 SEA	2012 total	2012 SEA	2013 total	2013 SEA	2014 total	2014 SEA
Dinoflagellates	8	0	12	0	17	0	2	0	2	0	9	0	19	0
Diatoms, other microalgae	1	0	0	0	0	0	3	0	0	0	4	0	0	0
Chlorophyta	4	0	6	0	8	0	3	0	2	0	5	0	13	0
Phaeophyceae	45	0	25	0	10	0	25	0	32	0	17	0	17	0
Rhodophyta	24	3	49	0	47	7	41	1	45	1	9	0	41	1

**Table 4:** Natural compounds isolated from different marine algae groups from Southeast Asia (data based on Blunt et al. 2016). The geographical origin and bioactivity of compounds are specified

Taxa	Compound	Bioactivity	Origin	Reference
*Gracilaria edulis* ^RH^	Levuglandin D_2_^n^	–	The Philippines	Kanai et al. 2011
*Laurencia nangii* ^RH^	Dihydroitomanallene B^n^ pannosallene^11^ itomanallene B^12^	–	Malaysia	Kamada and Vairappan 2012
*Laurencia snackeyi* ^RH^	5β-Hydroxypalisadin B^n, 13^	–	Malaysia	Wijesinghe et al. 2014
*Laurencia* sp.^RH^	Laurefurenyne A-F^n, 14,15,16^	cytotoxic	The Philippines	Abdel-Mageed et al. 2010
*Laurencia* sp.^RH^	Tiomanene^n^ acetylmajapolene A-B^n, 17^	–	Malaysia	Vairappan et al. 2008

*n* new compounds

RH rhodophyta

**Fig. 6:**
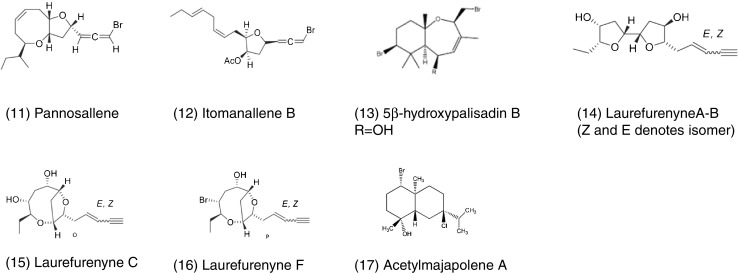
Antifouling compounds isolated from Southeast Asian macroalgae

^11–17^Compounds listed in Fig. 6

### Antifouling compounds from S.E.A

Although, studies looking at the antifouling activity of algae (micro and macro) are limited in S.E.A. compared to European and North American waters (Fig. 5), nevertheless studies over the last decade have shown promising antifouling activities from Southeast Asian algae. Majority of the studies from Southeast Asian waters have been largely focused on marine macroalgae, and antifouling activity was examined based on crude extract assays (e.g. Bhadury and Wright 2004 and references within; Sidharthan et al. 2004; Qian et al. 2010 and references within; Satheesh et al. 2016 and references within). Identified compounds so far from Southeast Asia with AF activity are provided in Fig. 6. An integrative approach involving identification of secondary metabolites from the crude extracts has been far less studied compared to information available from other geographical origins. This reflects the need to undertake approaches involving GC-MS and NMR for identification of potential potent antifouling secondary metabolites. While, Southeast Asia harbors high biodiversity including rich marine microalgae and macroalgal diversity, their potential for antifouling activity in an ecological context is yet to be effectively explored.

### Linking genes to biofilm formation

Among the early colonizers, diatoms play a significant role in biofilm development and are able to colonize on even most fouling-resistant surfaces (Molino and Wetherbee 2008). Currently, our understanding of the role of genes during bacteria-diatom interaction as part of biofilm formation is relatively well understood (e.g., Buhmann et al. 2012 and references within). However, limited knowledge exists in terms of the genes that are exclusively found in marine diatoms and their link to marine biofilm formation. The recent availability of phytoplankton genome sequence data, in particular diatom genomes, has resulted in improved understanding of mechanisms that control their growth and distribution in the marine environment (e.g., Armbrust et al. 2004; Bowler et al. 2008). Thus, the rapidly available diatom genome datasets can help in understanding the role of biosynthetic pathways towards production and regulation of extracellular polymeric substances (EPS) during the process of biofouling.

From the cellular viewpoint, the adhesive components of EPS are involved in diatom cell-substratum adhesion, in addition to motility. EPS molecules are secreted from frustules through pores in the girdle bands and valves. The carbohydrate components of the EPS have been characterized in various benthic diatoms (e.g., Chiovitti et al. 2008; Abdullahi et al. 2006). It is already well known that EPS secretion in diatoms depends on numerous factors including nutrient availability, daily fluctuations, irradiance, and even metal toxicity (Staats et al. 1999; Ai et al. 2015). This is particularly significant since EPSs can be a food source for heterotrophic organisms and affect the detachment of biofilms (e.g., De Brouwer and Stal 2002; Bellinger et al. 2005). For example, nitrogen and phosphate limitations affect production rate of EPSs including chemical composition in various diatoms (Magaletti et al. 2014; Ai et al. 2015). Mass spectrometry of EPSs of *Thalassiosira pseudonana* shows that degree of polymerization and distribution of EPSs can vary in response to nutrient depletion and different nutrient sources (Ai et al. 2015). Proteins and glycoproteins have been studied by chemical methods and by atomic force microscopy (Lind et al. 1997; Wustman et al. 1997; Dugdale et al. 2006; Chiovitti et al. 2008). It has been shown that the adhesive molecules appear to be highly glycosylated with novel glycans that are highly sulphated (Chiovitti et al. 2003, 2008).

**Fig. 7:**
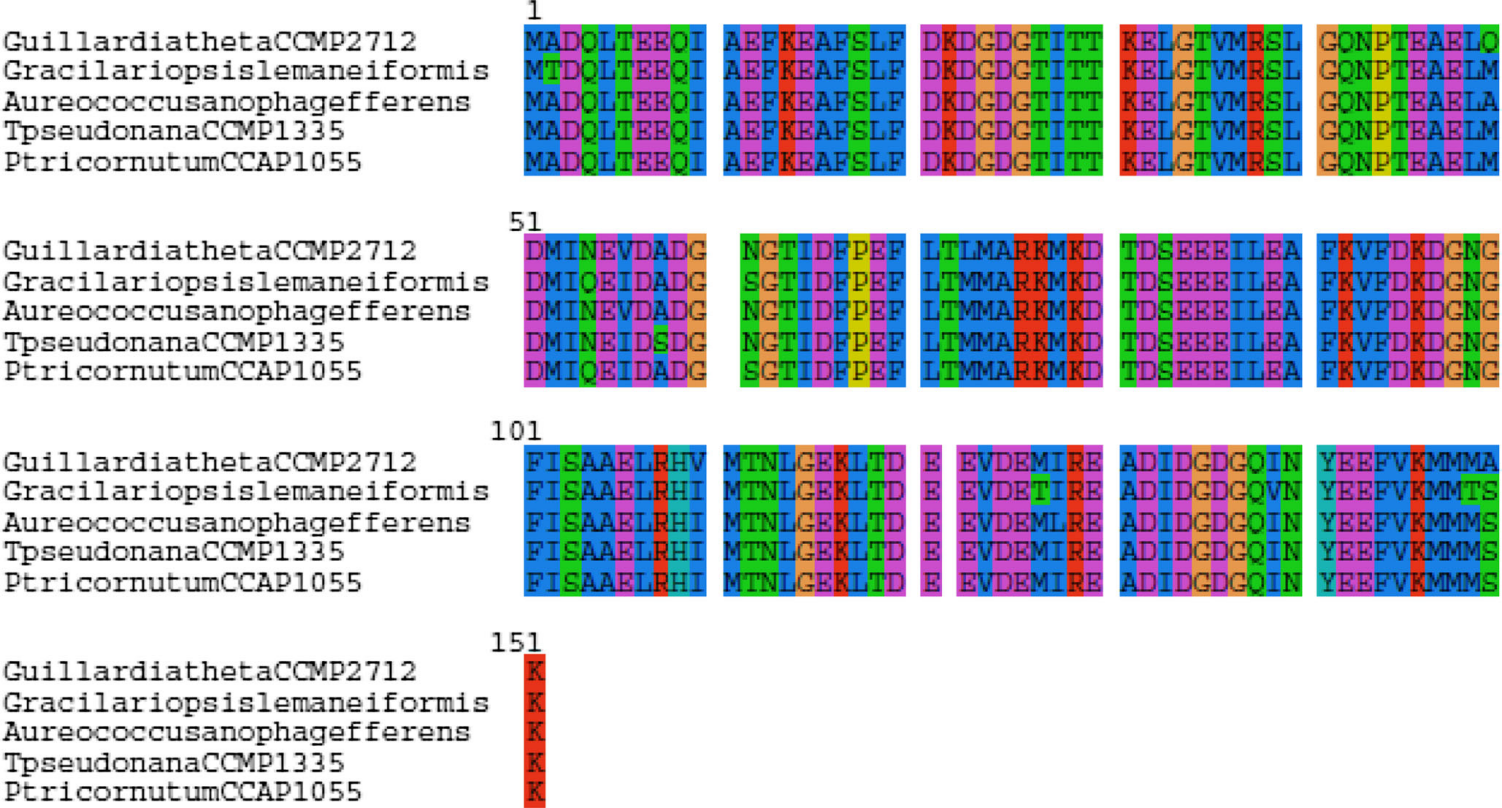
Amino acid sequence alignment of CAM gene from *Thalassiosira pseudonana* CCMP1335 (Acc No. XM_002295719), *Aureococcus anophagefferens* (Acc No XP_009033803), *Phaeodactylum tricornutum* CCAP1055 (Acc No. XP_002185049), *Guillardia theta* CCMP2712 (Acc No. XP_005836584) and *Gracilariopsis lemaneiformis* (Acc No. AKG55580) showing highly conserved regions

In a recent study, from the available genome dataset of the pennate diatom *Phaeodactylum tricornutum*, bioinformatic analysis was undertaken to identify putative diatom cell substratum adhesion molecules (PDC) (Willis et al. 2014). In total, 37 PDCs were identified from the *P*. *tricornutum* genome, of which some showed similarities to genes found in a diverse range of organisms, including metazoans, plants, and prokaryotes, as well as algae, encoding components of the extracellular matrix (ECM) or cell adhesion complexes (Yamada and Geiger 1997; Zhao and Waite 2006). It has been found that genes that code for PDCs have characterizing features that are common to a set of cell adhesion molecule (CAM) genes (Willis et al. 2014). Indeed, using bioinformatic approach, we detected the presence of CAM genes across sequenced marine micro- and macroalgal genomes (Fig. 7). As evident from Fig. 4, these amino acid sequences representing CAM are conserved across different marine algae and thus may also indicate the possible existence of putative PDCs in published marine algal genomes. Willis et al. (2014) reported that some of the PDCs were diatom specific and encoded unclear functions. These putative genes indicate that there is likely a diverse range of molecules that diatoms use for cell substratum adhesion and therefore these genes potentially may play key role in biofouling. Seven of these PDC have been characterized in vivo, by generation of transgenic diatom lines over-expressing genes encoding C-terminal yellow fluorescent protein (YFP) fusion proteins, and showed that these candidate proteins are involved in diatom cell adhesion. Based on the analysis of the *T. pseudonana* genome, several putative cell adhesion molecules have been identified using bioinformatics approach.

From the available transcriptome data of the model adhesion diatom, *Amphora coffeaeformis*, five proteins have been identified that exhibit unique amino acid sequences resembling the amino acid composition of the tyrosine-rich adhesion proteins from mussel footpads (Buhmann et al. 2014). Buhmann et al. (2014) looked into the function of one of these proteins, AC3362 by undertaking genetic transformation of *A*. *coffeaeformis*. They found that AC3362 plays a role in biosynthesis and or structural stability of the cell wall of this pennate diatom. The findings of AC3362 protein is particularly significant since in many mussel foot proteins, tyrosine residues have been post-translationally hydroxylated to 3,4-dihydroxyphenyl-L-alanine (Dopa) (Waite and Tanzer 1981). The presence of Dopa seems to play an important role in both structural integrities of the filaments and underwater adhesion to surfaces by forming covalent cross-links and coordination bonds with metal ions, as well as by forming hydrogen bonds with the surface. The AC3362 protein identified in *A*. *coffeaeformis* resembles the cingulins from the diatom *T. pseudonana*, which are also rich in tyrosine residues (Kröger and Poulsen 2008; Sumper and Brunner 2008). It will be therefore important to investigate using bioinformatics and experimental approaches the existence of similar homologs of this protein in other marine diatoms which are known to contribute to biofilm formation.

While the importance of EPS in biofilm formation is well known, other metabolic pathways are increasingly important in controlling diatom biofilm formation (e.g., Thompson et al. 2008). For example, it has been shown that a novel gene (PtNOA) linked to nitric oxide production (NO) along with its role in ribosome biogenesis and sporulation has been identified in *P*. *tricornutum* (Vardi et al. 2008). When this gene is over-expressed in transgenic diatoms, higher NO production is displayed and ultimately there is reduced ability to adhere to surfaces which is important in biofilm formation (Vardi et al. 2008). It is interesting to note that NOA-like sequences have been encountered in other marine diatoms and thus the role of this gene needs further investigation from the perspective of biofilm formation. Given that we are now starting to understand specific role of genes and proteins of microalgal origin in marine biofilm, the above information could help us to delineate basis of physiological complexity within a single microalgal species biofilm. Such morphological, physiological, and genetic basis of complexity is already under investigation with respect to prokaryotic single species biofilms (e.g., Archaea: Brazelton et al. 2011; Bacteria: Seth et al. 2012). Moreover, the identification of genes that implicitly regulates microalgal biofilm formation could form the basis for in-biofilm expression technology for understanding other microalgal-mediated biofilms as the above approach has been undertaken in case of bacterial biofilms (e.g., Finelli et al. 2003). Since diatoms play a crucial role in biofilm formation, it is essential therefore to undertake further identification of such genes, develop knockouts for disruption of biofilm, develop robust screening methodologies based on genetic data to identify stages of biofim, and ultimately strategize manipulation methods that could be used to biofouling under control from commercial viewpoint.

### Industrial scale-up of compounds production

Detection of antifouling potential from natural resources like macroalgae is not enough to meet the actual global demand. Thus, industrial scale up of novel antifouling compounds is a prerequisite. However, there are important limitations to industrially scale-up the production of antifouling metabolites from macroalgae. In general, extracting sufficient amounts of these metabolites may be extremely difficult due to limited quantity of the producer organism, the small amount of a specific compound within the target organism, or the variability of the concentration of these metabolites in response to biotic and abiotic factors. In consequence, the extraction of natural beds of seaweeds is not only ecologically destructive but it can also be unstable over time (Proksch et al. 2003; Pereira and Costa-Lotufo 2012), although large-scale extraction from drift algae may be possible (Siless et al. 2017). Given the limitations encountered with macroalgae, photosynthetic microorganisms are attracting considerable interest towards a sustainable production of natural products. This is due to their relatively high photosynthetic conversion efficiency, novel and diverse metabolic capabilities, faster growth rates, ability to thrive in diverse ecosystems, and ability to store or secrete energy-rich hydrocarbons (Radakovits et al. 2010). In theory, by using (cheap) sunlight and carbon dioxide (which is normally a waste), microalgae can produce high value metabolites of economic importance (such as antifouling metabolites). The potential productivity can be tenfold greater than that of agricultural crops and it can take place on non-arable land (Wijffels 2007). Thus, all these factors taken together makes them economically attractive and more environmentally friendly source of antifouling compounds.

Commercial large-scale culture of microalgae started in the early 1960s with *Chlorella*, and in a short period, the associated biotechnology industry has grown and diversified significantly (Spolaore et al. 2006; Borowitzka 2013b). These microorganisms are important sources of commercially produced high-value chemicals including carotenoids, long-chain polyunsaturated fatty acids, and phycobilins (Borowitzka 2013a). Markets of microalgae products already exist and are growing, but the growth of the markets is limited by the production technology used and cost-price of products, mostly limiting the commercial production to high-value products (Wijffels 2007). Among the main limitations for a successful algal industrial cultivation is the development of easy to use/low cost large operations systems, which also combine a sustainable use of water, temperature, light, nutrients, and gas resources, with limited microbial contamination, necessary to reduce the cost of production while maintaining and improving product quality (Courchesne et al. 2009; Ugoala et al. 2012).

Strain selection is also an important factor. So far, cultivation of microalgae has been limited to wild type strains of different species and bioprospecting has been used to isolate new strains with interesting and optimally combined properties (Hlavová et al. 2015). Microalgae are an extremely diverse group of organisms, which has not yet been fully explored in terms of diversity (Borowitzka 2013a). However, researchers are starting to believe that the most cost-effective way for industrial production lies in further improvements of current strains (Hlavová et al. 2015). It is believed that metabolic engineering is the way forward in case of large scale production of promising marine algal bioactive metabolites with antifouling potential (Bhadury and Wright 2004). For example, the possibility of using metabolic engineering approach to scale-up production of poly-unsaturated fatty acids has been explored in marine microalgae with some success (e.g., Courchesne et al. 2009, Khozin-Goldberg and Cohen 2011; see review by Mühlroth et al. 2013).

Metabolic engineering tools have been also used for overproduction of astaxanthin in microalgae by looking into the over-expression of PSY and CrtR-b genes in *Haematococcus pluvialis* (Chlorophyceae) (Li et al. 2008). Given that several marine algal genomes have been sequenced, such large-scale dataset has been also integrated as part of metabolic engineering tools. In the model marine diatom, *P. tricornutum*, intervention of metabolic engineering has enhanced accumulation of omega-3 long chain polyunsaturated fatty acids through generation of transgenic strains (Hamilton et al. 2014). Very recently, integration of flux balance analysis (FBA) and in silico proteomics has also shown promising results in terms of metabolic engineering application for sustainable microalgal energy development (e.g., Banerjee et al. 2016).

As mentioned, the industrial scale up of microalgae still faces several technological limitations which have to be considered for competing with commodities such as cuprous oxide, but we consider it important to explore the possibility of producing effective and more environmental friendly products. There is huge potential for application of metabolic engineering techniques for ultimate industrial scale-up of antifouling metabolites from marine microalgal origin. In theory, it offers not only the possibility to overcome “secondary production bottlenecks” but also to allow us to direct the production of secondary metabolites, or of important substrates for later synthesis of such antifouling compounds. Since establishment of transgenic microalgal strains has been (relatively) easy, a large production of metabolites with antifouling applications possibly can be achieved. This is supported by the increasing availability of genome level data generated from “omics”-based approaches which can be used as part of RNAi and riboswitch engineering approaches and may lead to enhanced production of antifouling metabolites. At the same time, it can be synchronized with rational biochemical engineering design for large-scale production of marine algal antifouling metabolites using photobioreactor technology. Given that there has been significant technological development in the field of metabolic engineering, one could expect that in coming years, this approach could prove to be useful for development of antifouling paints. However, the genetic improvement of algal strains is a current (moral and practical) challenge till now. Modified strains could overproduce traditional or newly discovered algal compounds and also serve to express specific genes that cannot be expressed into other organisms (Spolaore et al. 2006) and, therefore, produce different products under specific cultivation conditions. Radakovits et al. (2010) mentioned that more than 30 different strains of microalgae have been transformed successfully, but the use of transgenic microalgae for commercial applications has not been reported yet.

As new species are discovered and sequenced, and new tools become available for genetic manipulation, the rich diversity of microalgae can be exploited for new applications (Ruffing 2011). The possibility of producing a large amount of biomass in more environmentally friendly conditions offers higher chances for a successful industrial scale-up of the process. It is likely that such advances can be extended to industrially relevant microorganisms in the field of antifouling compounds.

## Conclusions

Given the evidence that has built up in the last few years reporting antifouling compounds extracted and isolated from marine algae—undoubtedly they continue to be a potential source for novel antifouling compounds with antibacterial, antibiofilm, antialgal, and anti-macrofouling properties. However, significant progress in terms of (a) fouling control in an environmentally friendly way and (b) successful exploitation of the reported active compounds is yet to be achieved and there is possibly still some way to go for full commercial use of such compounds. While we should still continue looking for such compounds in general and also exploit the untapped potential of S.E.A out of academic interests if not bioprospecting, we highlight the importance of (a) linking up biofilm formation with the diatom genes involved as identification of such genes could form the basis of future biofilm knockout mechanisms and (b) the need to put together screening, genomic, and metabolic engineering studies under one roof for successful interdisciplinary exploitation of such rich biodiversity of marine algal compounds.
