# Correction to: Minireview: algal natural compounds and extracts as antifoulants

**DOI:** 10.1007/s10811-018-1424-3

**Published:** 2018-05-01

**Authors:** Mahasweta Saha, Franz Goecke, Punyasloke Bhadury

**Affiliations:** 1 Benthic Ecology, Helmholtz Center for Ocean Research, Düsternbrooker weg, 24105 Kiel, Germany; 2 Present Address: School of Biological Science, University of Essex, Colchester, CO 43 SQ, UK; 3 Department of Plant and Environmental Science (IPV), Norwegian University of Life Sciences (NMBU), Ås, Norway; 4 Integrative Taxonomy and Microbial Ecology Research Group, Department of Biological Sciences, Indian Institute of Science Education and Research Kolkata, Mohanpur, Nadia, West Bengal 741246 India

**Correction to: J Appl Phycol**

10.1007/s10811-017-1322-0

The article “Minireview: algal natural compounds and extracts as antifoulants”, written by Mahasweta Saha, Franz Goecke, and Punyasloke Bhadury, was originally published electronically on the publisher’s internet portal (currently SpringerLink) on November 6, 2017 without open access.

With the author(s)’ decision to opt for Open Choice the copyright of the article changed on April 10, 2018 to © The Author(s) 2018 and the article is forthwith distributed under the terms of the Creative Commons Attribution 4.0 International License (http://creativecommons.org/licenses/by/4.0/), which permits use, duplication, adaptation, distribution and reproduction in any medium or format, as long as you give appropriate credit to the original author(s) and the source, provide a link to the Creative Commons license and indicate if changes were made.

The original article has been corrected.

